# Metabolism remodeling in pancreatic ductal adenocarcinoma

**DOI:** 10.15698/cst2019.12.205

**Published:** 2019-11-04

**Authors:** Jin-Tao Li, Yi-Ping Wang, Miao Yin, Qun-Ying Lei

**Affiliations:** 1 Fudan University Shanghai Cancer Center and Cancer Metabolism Laboratory, Institutes of Biomedical Sciences, Shanghai Medical College, Fudan University; Department of Oncology, Shanghai Medical College, Fudan University, Shanghai 200032, People's Republic of China.; 2 Lead Contact.

**Keywords:** PDAC, KRAS, metabolism, tumor microenvironment

## Abstract

Pancreatic ductal adenocarcinoma (PDAC) is predicted to become the second leading cause of death of patients with malignant cancers by 2030. Current options of PDAC treatment are limited and the five-year survival rate is less than 8%, leading to an urgent need to explore innovatively therapeutic strategies. PDAC cells exhibit extensively reprogrammed metabolism to meet their energetic and biomass demands under extremely harsh conditions. The metabolic changes are closely linked to signaling triggered by activation of oncogenes like *KRAS* as well as inactivation of tumor suppressors. Furthermore, tumor microenvironmental factors including extensive desmoplastic stroma reaction result in series of metabolism remodeling to facilitate PDAC development. In this review, we focus on the dysregulation of metabolism in PDAC and its surrounding microenvironment to explore potential metabolic targets in PDAC therapy.

## INTRODUCTION

**Figure 1:**
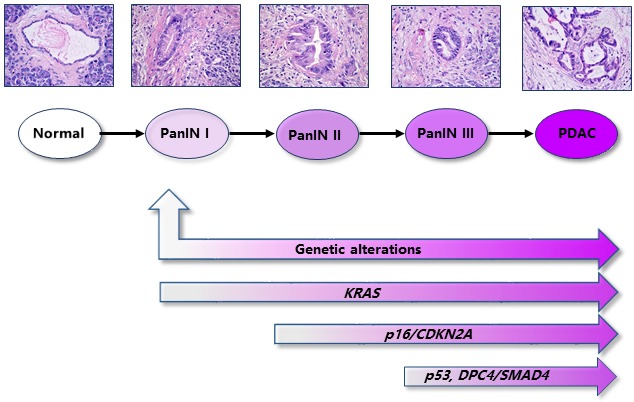
FIGURE 1: The pathological stages and dysregulated molecular events in PDAC development. The PDAC development process is categorized into pre-cancerous lesion (pancreatic intraepithelial neoplasia, PanINs) and malignant PDAC according to the pathological grade. Simultaneously, aberrant genetic alterations occurre at different stages of PDAC development, including activating mutations of *KRAS* and inactivation mutations of tumor suppressors, like *p16/CDKN2A, p53*, and *DPC4/SMAD4*.

Pancreatic ductal adenocarcinoma (PDAC) is a tumor with early metastatic potential and remarkable resistance to established therapies such as chemotherapy, radiotherapy, and molecular targeted therapy [1]. Given primary localization and cell morphology, PDAC has been generally viewed to be originated from pancreatic ductal cells. Nevertheless, dozens of studies including lineage tracing propose alternative original sources for PDAC cells. For example, acinar-to-ductal metaplasia (ADM) frequently occurres at the early stage of pancreas carcinoma, suggesting an acinar origin of PDAC [2–5]. In addition, emerging evidences suggest that some rare cell population named as cancer stem cell might be the precursor of PDAC cells for initiation and metastasis [1, 6]. Malignant progression of PDAC, starting from the pre-cancerous lesion, pancreatic intraepithelial neoplasia (PanINs), to advanced invasion and metastasis is accompanied by various oncogene activation and tumor suppressor inactivation. *KRAS, p16/CDKN2A, TP53,* and *DPC4/SMAD4* are viewed as driver genes in PDAC development because of their high frequency of mutation during tumorigenesis of PDAC [4]. *KRAS* activating mutations occur at early PanIN I stage in 95% of cases, followed by the loss of the functional tumor suppressor gene *p16/CDKN2A* (> 90%). Inactivating mutations in *TP53* (75%) and *DPC4/SMAD4* (55%) are often observed at late PanIN III stage as shown in **Figure 1** [4, 7].

Nutrients in the form of carbohydrates, amino acids, and fatty acids are utilized to support biosynthesis, supply energy and balance oxidative stress. Aberrant alterations of signaling rewire intermediary metabolism to support energetic and biosynthetic demands of PDAC cells. Of note, PDAC cells are surrounded by a microenvironment which is composed of immune cells, stellate cells, and extracellular matrix (ECM) [8, 9]. It is therefore necessary to expand our knowledge on the effect of metabolic remodeling of the tumor microenvironment on PDAC development.

## METABOLIC REPROGRAMMING IN RESPONSE TO ABNORMAL SIGNALING IN PDAC CELLS

**Figure 2:**
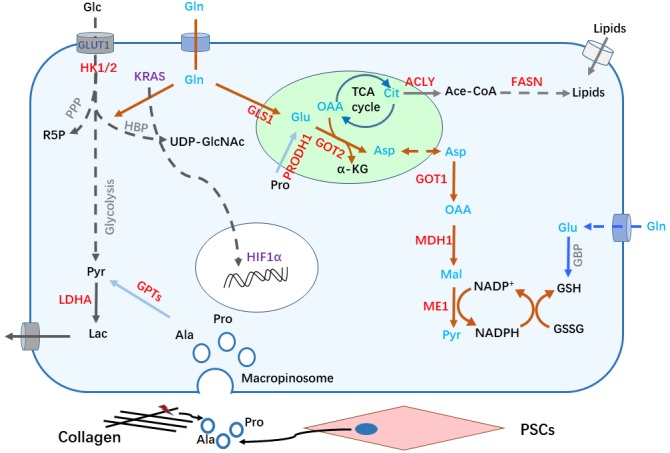
FIGURE 2: Metabolic remolding in PDAC cells. **(1)** KRAS (in cytoplasm) and HIF1α activation in PDAC cells upregulates glucose transporter (GLUT1) and other glycolytic related genes to promote glucose (Glc) uptake and enhance glycolysis flux, including the production of lactate (Lac) and carbon donation into the hexosamine biosynthetic pathway (HBP) and pentose phosphate pathway (PPP). **(2)** KRAS activation reprograms Gln metabolism to balance cellular redox homeostasis. Gln is sequentially converted to Glu and Asp catalyzed by GLS1 and GOT2 in the mitochondria, Asp is shuttled to the cytoplasm and generates NADPH after a series of reactions to maintain redox homeostasis. **(3)** The lipid synthesis pathway is activated, citrate is shuttled from the mitochondria into the cytoplasm to produce acetyl CoA (Ace-CoA), thereby enhancing *de novo* lipid synthesis pathway. Concomitantly, uptake of exogenous lipids is increased to meet the demand of nutrients for rapid proliferation. **(4)** The tumor microenvironment, including ECM components and stromal cells, also provide various metabolites/nutrients for PDAC cells, such as Ala and Pro derived from collagen degradation or pancreatic stellate cells (PSCs) secretion. The gene expression of molecules labeled in red color are up-regulated. α-KG: alpha ketoglutarate; Ace-CoA: acetyl-Coenzyme A; ACLY: ATP-citrate lyase; Ala: alanine; Asp: aspartate; Cit: citrate; FASN: fatty acid synthase; Glc: glucose; Gln: glutamine; GLS1: glutaminase; Glu: glutamate; GOT: glutamic-oxaloacetic transaminase; GPT: glutamic pyruvic transaminase; GSH: glutathione reduced; GSSG: glutathione oxidized; HBP: hexamine biosynthetic pathway; HIF: hypoxia-inducible factor; Lac: lactate; LDHA: lactate dehydrogenase A; MDH: malate dehydrogenase; ME: malic enzyme; OAA: oxaloacetic acid; PPP: pentose phosphate pathway; Pro: proline; PRODH1: proline dehydrogenase; PSC: pancreatic stellate cell; Pyr: pyruvate; R5P: ribose-5-phosphate; TCA: tricarboxylic acid.

Tumor cells prefer to take up more glucose for glycolysis even in presence of abundant oxygen, which is known as Warburg effect [10]. Constitutive activation of *KRAS*, the most prevalent genetic alteration in PDAC development, plays a key role in metabolic reprogramming, particularly in the glycolytic switch [11–14]. Analyses of gene expression and metabolic flux show that oncogenic KRAS upregulates expression of glucose transporter -1(GLUT1) to increase glucose influx, and hexokinase (HK) 1 and 2 to speed up glycolytic activity [13, 15]. Enhanced glycolysis driven by oncogenic *KRAS* supports biomass synthesis. The hexosamine biosynthetic pathway (HBP), a side path of glycolysis, is driven by *KRAS* mutation to provide precursors for protein glycosylation **(Figure 2)** [15, 16]. In addition, KRAS activation leads to enhanced entry of glucose carbon into the pentose phosphate pathway (PPP) [15]. PPP-derived ribose-5-phosphate (R5P) provides materials for DNA and RNA synthesis in proliferating cells. Generally, PPP is divided into two phases: oxidative and non-oxidative. Pancreatic cancer cells with KRAS mutation become dependent on non-oxidative PPP. Consistently, *KRAS* knockdown decreases expression of enzymes that govern non-oxidative PPP flux, resulting in strong growth inhibition [15]. Of note, most normal cells generate R5P through oxidative PPP. This discrepancy represents a potential metabolic vulnerability in *KRAS*-driven PDAC [17]. Moreover, *KRAS* activation coordinates with *p16* ablation to upregulate the expression and enzymatic activity of NAD(P)H oxidase 4 (NOX4), leading to enhanced oxidation of NAD(P)H. NAD^+^, the catalytic product of NAD(P)H, significantly potentiates glycolysis [18].

Glutamine (Gln) belongs to the group of non-essential amino acid (NEAA) that contribute to nitrogen and carbon donation for rapidly proliferating cells. Besides, *KRAS* mutant PDAC cells become addicted to Gln. *KRAS* directs an alternative metabolic pathway of Gln by transcriptionally regulating expression of key metabolic enzymes including glutamate dehydrogenase (GLUD1) and aspartate transaminase (GOT1), maintaining redox balance in PDAC cells. In this non-canonical pathway, Gln-derived carbon is converted into aspartate (Asp) through a series of reactions in mitochondria. Gln-derived Asp is then released into the cytoplasm and converted to oxaloacetic acid (OAA) and malate, eventually producing NADPH, a reducing equivalent involved in biosynthesis and reduction of reactive oxygen species (ROS) **(Figure 2)** [19]. Additionally, *Nrf2*, known as ROS suppressor, facilitates PDAC development [20]. The expression levels of *Nrf2-*regulated anti-oxidant genes are induced by *KRAS* [21]. Therefore, low intracellular ROS levels caused by *KRAS* signaling is vital for PDAC development.

*p53* mutation is another prominent genetic event in PDAC development. In contrast to *KRAS* mutations which occur at early stage to initiate PanIN lesions, mutations in *p53* are frequently present at late stages and substantially promote PDAC progression. *p53* mutation was reported to prevent the glycolytic enzyme glyceraldehyde-3-phosphate dehydrogenase (GAPDH) from nuclear translocation and increased GAPDH stability in the cytoplasm, supporting glycolysis to avoid apoptosis and autophagy [22]. In addition, *p53* mutations result in a decrease of intermediates in the tricarboxylic acid (TCA) cycle, indicating mitochondrial repression in PDAC cells [23]. Recent work demonstrated that differentiation of malignant PDAC cells could be triggered to prevent PDAC development by p53-dependent increase of α-ketoglutarate (αKG) [24].

Hypoxia commonly occurs in tumor progression, which induces the expression of hypoxia-inducible factor-1 (HIF-1) and increases its stability. Upregulation of HIF-1α could be detected in PDAC. In preclinical models, hypoxia inducible GFPT2 isoform to accelerate HBP [25]. In addition, hypoxia-inducible factor 1a (HIF1a) upregulates GLUT1 as well as the expression of other glycolysis-related genes to generate cytosolic ATP in PDAC cells **(Figure 2)** [26–28]. However, the effect of HIF1a in PDAC development may be context-dependent. One study showed that HIF1a invalidation supports PDAC formation in a mouse model [29].

The hippo pathway was originally identified to function in development, such as controlling organ size [30]. After that, the role of the Hippo pathway in cancer development has been investigated [31]. Particularly, recent studies indicate that Yap1/Tead2 promote PDAC in a KRAS-independent manner [32]. Moreover, accumulating evidences show the mutual interaction between the Hippo pathway and different metabolic pathways [33]. It was reported that glucose metabolism, amino acid metabolism, lipid metabolism and mitochondrial fusion are regulated by YAP and TAZ [34]. YAP phosphorylation and nuclear translocation are modulated by different stress signals and various nutrients/metabolites, including redox status, glucose, lipids, and G protein-coupled receptors (GPCRs). Reciprocally, Hippo signaling also fine-tunes metabolism in the body. Activation of YAP/TAZ upregulates expression of genes encoding both transporters to increase uptake of nutrients/metabolites and rate-limiting enzymes to promote glycolysis and glutamine catabolism [34]. Therefore, it is of great interest to define the effect of Hippo pathway on metabolism reprogramming in PDAC development.

In addition to the dysregulation of glucose and Gln metabolism, adaptive changes of various nutrient/metabolites in PDAC development have been uncovered. Preliminary data indicate that decrease of arginine levels could trigger pancreatic cancer cells death. Some clinical trials of bacterial arginine deaminase or bioengineered arginase, aiming to reduce arginine levels, are undergoing in patients with different cancer [35, 36]. 5-hydroxytryptamine (5-HT) is known as neurotransmitter which controls critical cognitive and behavioral functions of humans. A recent report demonstrated that 5-HT is elevated in PDAC cells, accompanied by increased tryptophan hydroxylase (TPH1) and decreased mitochondrial enzyme monoamine oxidase A (MAOA) to regulate 5-HT synthesis pathway and degradation pathway, respectively [37]. In addition, treatment with 5-HT receptor inhibitor suppresses growth and reprograms metabolism of pancreatic tumors, prolonging the survival of *KPC* mice [37]. Moreover, 5-HT activates small GTPase Ras-related C3 botulinum toxin substrate 1 (Rac1) to induce trans-differentiation of acinar cells into ductal, known as ADM [38].

## LIPID METABOLISM IN PDAC

To sustain uncontrolled cell proliferation, cancer cells need to keep generating various cellular components. Lipids are fundamental materials for structures of cells. Emerging evidence indicates that activated lipid synthesis is required for cancer cell growth. At the initial step of *de novo* lipid synthesis, ATP-citrate lyase (ACLY) converts citrate to cytoplasmic acetyl-CoA, followed by conversion to malonyl-CoA by acetyl-CoA carboxylase (ACC). Acetyl-CoA and malonyl-CoA are coupled to acyl-carrier protein (ACP) domain of fatty acid synthase (FASN) in an NADPH-dependent manner to synthesize palmitic acid (16-carbon saturated fatty acid) **(Figure 2)** [39].

Lipogenic enzymes including ACLY are frequently overexpressed in PDAC [40] [41]. The growth of PDAC cells is inhibited by the interference of ACLY activity in a xenograft tumor model [42]. In addition, patients with pancreatic cancer expressing high levels of FASN display shorter overall survival period than patients with low FASN expression [43].

Cholesterol is an essential structural component of cell membranes. Fatty pancreases were observed in human and are associated with increased risk of pancreas cancer [44]. Furthermore, expression of HMG-CoA (3-hydroxy-3-methylglutaryl-Coenzym-A) reductase and LDLR (low density lipoprotein receptor) is elevated in *KRAS*-driven PDAC mouse model. Consistently, LDLR silencing reduces ERK signaling activity and inhibits PDAC cell proliferation [45].

Hypoxia-inducible HIF-1 also supports lipid synthesis. HIF-1 activation suppresses αKG dehydrogenase (αKGDH), which drives the metabolic shift from the TCA cycle to IDH (isocitrate dehydrogenase)-mediated FA (fatty acid) synthesis [46]. Our study demonstrated that acetate functions as an epigenetic metabolite to promote *de novo* lipid synthesis under hypoxia conditions [47]. Those data broaden our scope to explore additional function of lipid metabolism in PDAC development.

## AUTOPHAGY, MITOCHONDRIA, AND PANCREAS CANCER

Classical autophagy is initiated from the formation of a membrane-structured autophagosome which transports damaged cellular components to the lysosome when cells receive stressful stimuli, such as hypoxia, starvation, chemotherapy, and radiation. Processed materials in lysosomes are degraded or recycled to keep cell homeostasis. In tumors, autophagy has an anti- or pro-cancerous function. Enhanced autophagy is detected in PDAC. Nevertheless, opposite evidence shows that elimination of autophagy contributes to PDAC initiation [48]. One interesting study found that KRAS disruption augments autophagy in PDAC [49]. Moreover, our study revealed that acetylation at the K5 residue of lactate dehydrogenase A (LDHA) triggers chaperone-mediated autophagy (CMA) and delivers acetylated LDHA to lysosome for degradation, resulting in alteration of intracellular lactate flux and alleviating the malignant phenotype of PDAC cells *in vivo* and *in vitro* [50]. These sophisticated functions of autophagy may explain the poor response to chemotherapy and radiotherapy in current PDAC treatment.

Over the past two decades, targeted therapy is one of the most important advances in the treatment of patients with cancer. However, targeting *KRAS* in PDAC obtains disappointing results. One explanation may be due to the “undruggable” structure of RAS while some recent studies reveal promising advance of RAS inhibitor development [51]. On the other hand, it has been identified that a small population of PDAC cells characterized by stem cell features depend on mitochondrial oxidative phosphorylation and lose response to KRAS signaling [52]. In addition, as mitochondrial respiration is the major resource of intracellular ROS, our study demonstrated that methylation at R248 of malate dehydrogenase 1 (MDH1) is essential to maintain cellular redox homeostasis in PDAC cells [53]. Notably, a novel mechanism that KRAS activation stimulates mitophagy via NIX to sustain PDAC development is disclosed [54]. Altogether, these results indicate that targeting mitochondrial respiration and/or KRAS signaling would significantly improve treatment efficiency of PDAC [51–53].

## METABOLIC REMODLING OF THE PDAC MICROENVIRONMENT

Studies in the recent two decades show that abnormal metabolism remodeling in the tumor microenvironment largely contributes to the poor survival of patients with PDAC.

Collagens are the most enriched ECM molecules in the PDAC tumor microenvironment. PDAC cells could uptake cleaved collagen fragments or collagen-derived proline (Pro) through a macropinocytosis-dependent or -independent process **(Figure 2)**. Engulfed collagen fragments are degraded to produce free amino acids in the lysosome, which are entering the TCA cycle and are further metabolized to supply building blocks to promote PDAC cell survival [55]. A further report showed that stimulation of epidermal growth factor receptor (EGFR) - Pak enhances micropinocytosis in PDAC upon nutrient stress [56].

Multiple types of stromal cells reside in the PDAC microenvironment. Among them, pancreatic stellate cells (PSCs) are tissue-specific fibroblasts within the pancreas. Reciprocal regulation between PSCs and PDAC cells has been intensively investigated. PSCs-secreted leukemia inhibitory factor (LIF) promotes malignancy of PDAC cells via paracrine [57]. Cancer-associated PSCs also secretes alanine (Ala) to feed PDAC cells and fuel TCA cycle, providing alternative nutrients for cancer cells and decreasing their addiction to glucose as well as other serum-derived nutrients in the austere tumor microenvironment **(Figure 2)** [8, 58, 59]. Intriguingly, vitamin D and all-trans retinoic acid (ATRA) were reported to revert stellate cells to a quiescent state, suppressing matrix remodulation and inhibiting cancer cell invasion [60, 61].

Cancer has been viewed as chronic inflammation without healing. Indeed, various inflammatory cells are involved in tumorigenesis. Tumor-associated macrophages (TAMs) have been reported to promote cancer development. Interestingly, increased glycolysis is observed in TAMs [62]. Furthermore, disruption of PI3Kγ acting as key lipid kinase in macrophages significantly abrogates PDAC invasion and metastasis by enhancing CD8^+^ T-cell immunosuppression [63]. It is noteworthy that ablation of HIF1a in the pancreatic tissue dramatically boosts malignant progress of *Kras*^G12D^-driven PanIN by recruiting a specific subgroup of B cells to infiltrate into the tumor microenvironment [29].

Based on these findings, therapeutic strategies against the tumor microenvironment are becoming an attractive opportunity to beat this lethal disease.

## CONCLUSIONS AND PROSPECTS

To adapt severely metabolic constraints, PDAC cells rely on specific metabolic reprogramming, offering potentially innovative strategies to treat patients with PDAC in the future. It was reported that PDAC cells could be divided into three different subtypes according to their metabolic profiling, including slow proliferating, glycolytic, and lipogenic subtypes **(Table 1)** [64]. The glycolytic subtype is more sensitive to glycolytic and glutamine inhibitors, while the lipogenic subtype is more sensitive to inhibitors of lipid biosynthesis. The metabolic plasticity greatly contributes to cancer heterogeneity. A further study on cancer heterogeneity in PDAC revealed that ductal cells are divided into two types based on the features of gene expression profiles via single-cell RNA-sequence analysis in PDAC [65]. Type 1 ductal cells are relative normal and present in both normal and cancer tissues, while type 2 ductal cells are exclusively found in the PDAC region, furthermore, type 2 ductal cells also contain seven subpopulations. Meanwhile, based on single-cell RNA-sequence analysis stromal cells including T cells, macrophages and fibroblasts are highly heterogeneous within the tumor microenvironment? [65, 66]. Therefore, targeting cancer metabolism in combination with other targeting agents or cytotoxic compounds would be promising therapeutic strategies beneficial for PDAC patients.

**Table 1.** Subtypes of PDAC based on metabolic features.

**Subtype**	**Metabolic features**	**Therapeutic strategies**
Slow proliferating	Low levels of amino acids and carbohydrates	Glycolytic inhibitors or inhibitors of lipid biosynthesis
Glycolytic	Metabolites elevated in glycolytic and serine pathways, lower levels of metabolites of redox homeostasis	Glycolytic, Gln inhibitors and ROS-inducing agents
Lipogenic	Enriched for lipid metabolites and TCA cycle metabolites	Inhibitors of lipid biosynthesis

## Abbreviatons:

5-HT: – 5-hydroxytryptamine,
αKG: – alpha-ketoglutarate,
ADM: – acinar-to-ductal metaplasia,
Asp: – aspartate,
ACLY: – ATP-citrate lyase,
ECM: – extracellular matrix,
GAPDH: – glyceraldehyde-3-phosphate dehydrogenase,
Gln: – glutamine,
HBP: – hexosamine biosynthetic pathway,
HIF: – hypoxia-inducible factor,
LDH: – lactate dehydrogenase,
LDLR: – low density lipoprotein receptor,
PanIN: – pancreatic intraepithelial neoplasia,
PDAC: – pancreatic ductal adenocarcinoma,
PPP: – pentose phosphate pathway,
PSC: – pancreatic stellate cell,
R5P: – ribose-5-phosphate,
ROS: – reactive oxygen species,
TAM: – tumor-associated macrophage,
TCA: – tricarboxylic acid cycle.
